# The Q-LAMP Method Represents a Valid and Rapid Alternative for the Detection of the *BCR-ABL1* Rearrangement in Philadelphia-Positive Leukemias

**DOI:** 10.3390/ijms20246106

**Published:** 2019-12-04

**Authors:** Stefania Stella, Enrico Marco Gottardi, Valeria Favout, Eva Barragan Gonzalez, Santa Errichiello, Silvia Rita Vitale, Carmen Fava, Luigia Luciano, Fabio Stagno, Francesco Grimaldi, Lucrezia Pironi, Claudia Sargas Simarro, Paolo Vigneri, Barbara Izzo

**Affiliations:** 1 Department of Clinical and Experimental Medicine, University of Catania, 95123 Catania, Italy; silviarita.vitale@gmail.com (S.R.V.); vigneripaolo@gmail.com (P.V.); 2 Center of Experimental Oncology and Hematology, A.O.U. Policlinico-Vittorio Emanuele, 95123 Catania, Italy; 3 Department of Clinical and Biological Sciences, University of Turin, A.O.U. San Luigi Gonzaga, 10043 Orbassano (Turin), Italy; enricogottardi@libero.it (E.M.G.); valeria.favout@gmail.com (V.F.); carmen.fava@unito.it (C.F.); lucrezia.pironi@yahoo.it (L.P.); 4 Molecular Biology Unit, La Fe Universitary and Polytechnic Hospital, La Fe Health Research Institute, 46000 Valencia, Spain; barragan_eva@gva.es (E.B.G.); Claudia_sargas@iislafe.es (C.S.S.); 5 Centro Ingegeneria Genetica (CEINGE)–Biotecnologie Avanzate, University Federico II, 80131 Naples, Italy; errichiello@ceinge.unina.it; 6 Hematology Unit, A.O.U. Federico II, University of Naples, 80131 Naples, Italy; lulucian@unina.it (L.L.); grimaldi.francesco@gmail.com (F.G.); 7 Division of Hematology and Bone Marrow Transplant, AOU Policlinico-Vittorio Emanuele, 95123 Catania, Italy; fsematol@tiscali.it; 8 Department of Molecular Medicine and Biotechnology, University Federico II, 80131 Naples, Italy; barbara.izzo@unina.it

**Keywords:** chronic myeloid leukemia, *BCR-ABL1*, Q-LAMP, e13a2, e14a2, rare transcripts

## Abstract

Molecular detection of the *BCR-ABL1* fusion transcripts is necessary for the genetic confirmation of a chronic myeloid leukemia diagnosis and for the risk classification of acute lymphoblastic leukemia. *BCR-ABL1* mRNAs are usually identified using a conventional RT-PCR technique according to the BIOMED-1 method. In this study, we evaluated 122 *BCR-ABL1*-positive samples with the Q-LAMP assay to establish if this technology may represent a valid alternative to the qualitative BIOMED-1 PCR technique usually employed for the detection and the discrimination of the common *BCR-ABL1* transcripts (p190 and p210 isoforms). We found a 100% concordance rate between the two methods. Specifically, the p190- and p210-positive samples were amplified by Q-LAMP with a median threshold time (Tt) of 26.70 min (range: 24.45–31.80 min) and 20.26 min (range: 15.25-34.57 min), respectively. A median time of 19.63 was observed in samples displaying both (e13a2/e14a2) p210 isoforms. Moreover, the Q-LAMP assay allowed recognition of the *BCR-ABL1* e13a2 and e14a2 isoforms (median Tts 18.48 for e13a2 vs. 26.08 min for e14a2; *p* < 0.001). Finally, 20 samples harboring rare *BCR-ABL1* isoforms (e1a3, e13a3, e14a3, and e19a2) were correctly identified by the Q-LAMP assay. We conclude that the Q-LAMP assay may represent a faster and valid alternative to the qualitative BIOMED-1 RT-PCR for the diagnosis at *BCR-ABL1*-positive leukemias, especially when samples are analyzed in centers with restricted resources and/or limited technical expertise.

## 1. Introduction

The Philadelphia (Ph) chromosome is the first cytogenetic marker associated with a malignant neoplasia as it is present in 95% of chronic myeloid leukemia (CML) cases, in 3-5% of pediatric acute lymphoblastic leukemia (ALL), and in 15%–20% of adult ALL [1,2,3].

The Ph chromosome is generated by a reciprocal translocation between the long arms of chromosomes 9 and 22 t(9;22) (q34;q11) with the *ABL1* oncogene juxtaposed to the breakpoint cluster region (*BCR*) gene on chromosome 22 [4]. This cytogenetic alteration gives rise to the *BCR-ABL1* chimeric gene encoding for an oncoprotein with constitutive tyrosine kinase (TK) activity that alters the proliferation rates, survival signaling, immunological interactions, and cytoskeleton dynamics of hematopoietic stem cells [5,6,7,8,9]. The breakpoint in the *BCR* gene is most frequently located downstream of exon 13 or exon 14 (e13 and e14, previously referred to as exons b2 and b3), in the major cluster region (M-BCR), while the most common breakpoint in the *ABL1* gene is upstream of exon 2 (a2). These breakpoints lead to the e13a2 or e14a2 *BCR-ABL1* rearrangements, which encode for a 210 kDa protein called p210. In about 75% of *BCR-ABL1*-positive ALL, but <1% of CML patients, the breakpoint in *BCR* occurs between exons 1 and 2 in the microcluster region (µ-BCR), generating the e1a2 mRNA fusion that is translated into the p190 oncoprotein. In about 2–3% of CML patients, a breakpoint downstream of *BCR* exon 19 results in an e19a2 fusion transcript that gives rise to a 230 kDa protein (p230). Other infrequent breakpoints involve *BCR* exons 6 or 8 (e6a2 or e8a2) or *ABL1* exon 3 (e13a3 or e14a3) [1,10,11,12,13,14].

Several studies have investigated whether the quantity or quality of the transcript type may influence the outcome of *BCR-ABL1*-positive patients [10,15,16,17,18,19]. Previous evidence has suggested that the type of transcript may impact the response to tyrosine kinase inhibitors (TKIs) [3,20]. Indeed, Jain et al. reported that CML patients with e13a2 display a trend towards inferior event-free (EFS) and transformation-free survival (TFS) compared to the e14a2 isoform (alone or co-expressed with e13a2) [10]. Moreover, in the imatinib (IM) era, some studies suggested that the e14a2 fusion was associated with faster and deeper responses [15,16,17].

Currently, identification of *BCR-ABL1* transcripts is routinely performed to confirm a CML or ALL diagnosis and allow the timely assignment of an appropriate treatment regimen for each patient [21,22,23,24].

In the last 15 years, several qualitative and quantitative molecular techniques have been developed to identify and measure *BCR-ABL1* transcripts. With the introduction of real-time quantitative polymerase chain reaction (RT-qPCR), serial measurements of *BCR-ABL1* mRNA can be performed to monitor patient outcomes and, if necessary, reassess the assigned therapy. The methodology used to identify the *BCR-ABL1* fusion has evolved over the years. The most common molecular diagnostic method is based on a conventional RT-PCR following the BIOMED-1 protocol, a multistep procedure that identifies the translocations and defines the breakpoints [25].

An easier and faster molecular assay for *BCR-ABL1*-positive leukemias may improve patient management and should ideally be feasible in clinical laboratories located outside of a specialized center, such as small hospitals or laboratories in developing countries.

The loop-mediated isothermal amplification (LAMP) technology is a non-PCR-based nucleic acid amplification method that rapidly amplifies DNA or RNA targets under isothermal conditions. LAMP is performed using a strand-displacement polymerase and does not require Taq DNA polymerase or thermal cycling [26]. The potential diagnostic applications of this technology are remarkable and rapidly expanding, particularly in the field of infectious [27,28,29] and hematologic diseases [30,31,32]. The Q-LAMP assay (DiaSorin S.p.A., Saluggia, Italy) represents a technical improvement over the classical LAMP resulting in a more sophisticated method suitable for the needs of oncohematology diagnoses.

In this study, we evaluate if the Q-LAMP technology may represent a valid alternative to the BIOMED-1 PCR method for the detection and the discrimination of the common *BCR-ABL1* transcripts in Ph-positive leukemia patients. Moreover, we investigate if the Q-LAMP method is potentially able to detect rare *BCR-ABL1* isoforms.

## 2. Results

### 2.1. Concordance between the Q-LAMP Assay and the Standard BIOMED-1 Method on p210 and p190 BCR-ABL1 Isoforms

**Figure 1:**
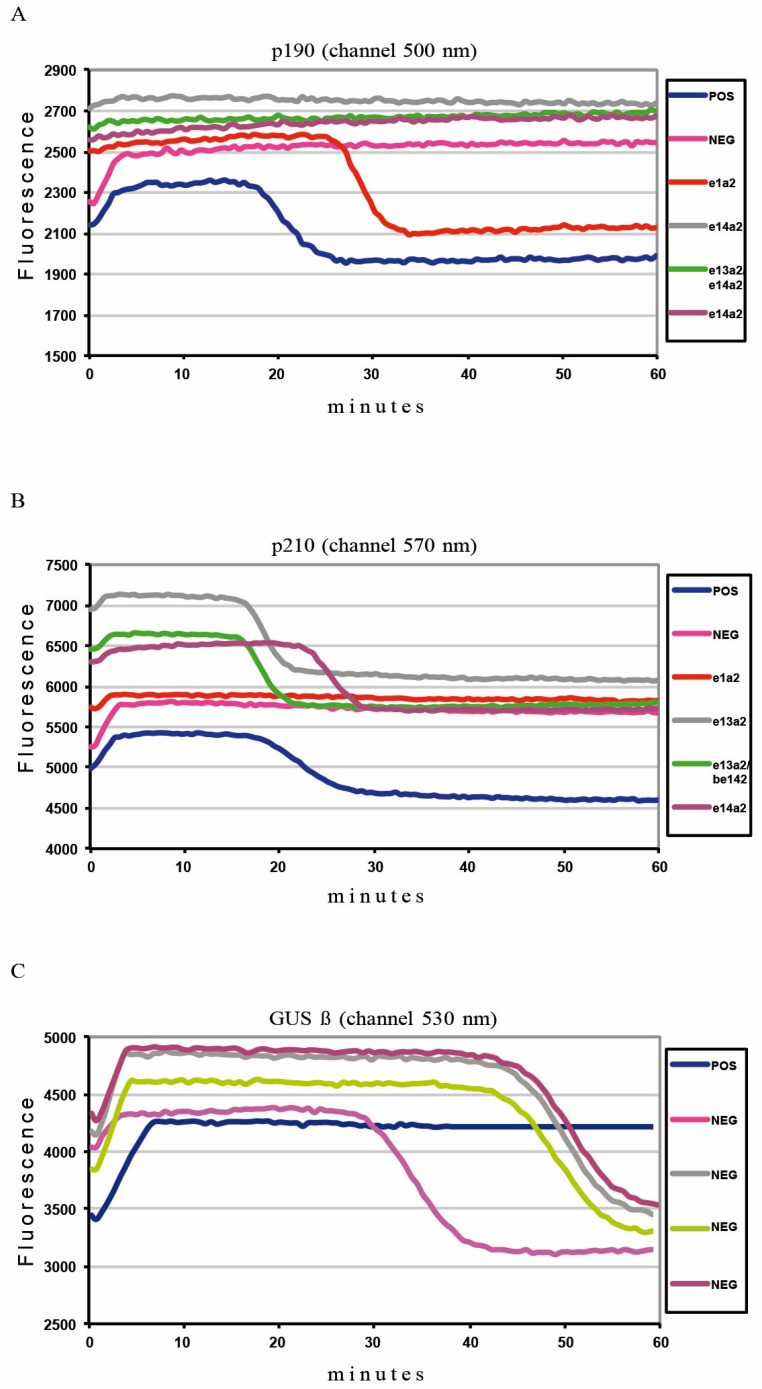
*BCR-ABL1* Q-LAMP amplification plot. Representative fluorescence quenching curves for *BCR-ABL1* p190 (panel **A**, red curve) and for *BCR-ABL1* e13a2 (panel **B**, gray curve), e14a2 (panel B, violet curve) or e13a2/e14a2 (panel B, green curve). In the negative specimen, there is amplification of the housekeeping control *GUSß* gene (panel **C**, pink curve).

In order to evaluate the Q-LAMP assay specificity we analyzed a total of 122 *BCR-ABL1*-positive samples (102 positive for p210 and 30 for p190) and 50 negative samples (24 derived from *BCR-ABL1*-negative patients and 26 from healthy human donors), as previously described by Salmoiraghi et al. [33]. The obtained data were compared to results achieved with the RT-PCR following the BIOMED-1 protocol. We found a 100% concordance rate between the two methods (Table 1). Both p190- and p210-positive samples were identified by Q-LAMP in less than an hour with visible amplification status at median Tt of 26.70 min (range: 24.45-31.80) and 20.26 min (range: 15.25–34.57), respectively (Figure 1A,B and Table 2). All negative samples were validated through amplification of the housekeeping *GUSß* control gene with a median Tt of 41.94 min (range: 38.03–51.77) (Figure 1C). 

### 2.2. Q-LAMP Performance on Common BCR-ABL1 p210 Isoforms

**Figure 2:**
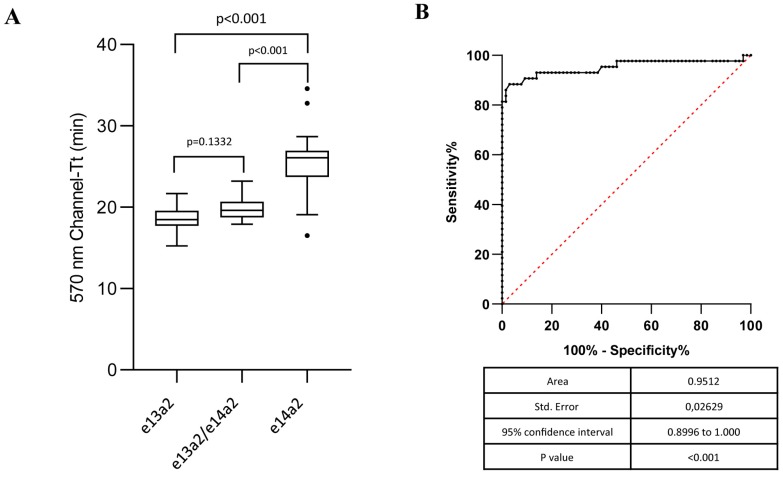
Q-LAMP performance on common *BCR-ABL1* p210 isoforms. (**A**) Median threshold times (Tts) in minutes were determined for each group (e13a2, e13a2, and e13a2/e14a2 samples) and depicted as boxplots delimited by the 25th (lower) and 75th (upper) percentile. Horizontal lines above and below each boxplot indicate the 5th and 95th percentile, respectively. Thick lines in each boxplot represent median Tts in each patients group. *p* values refer to statistical significance among the groups indicated by the bracket. The distribution of the e14a2 Tt is significantly different compared to the e13a2 or the e13a2/e14a2 groups (*p* < 0.001). (**B**) The ROC indicates the sensitivity, specificity, and cutoff value of the different Tts.

We next investigated a possible correlation between the Tt and the different transcript variants. We stratified patients according to their isoforms (e1a2, e13a2, e14a2, and both e13a2/e14a2) and then compared their Tts (Table 2). Interestingly, our statistical analysis demonstrated that the e13a2 and e14a2 isoforms showed different Tts with a visible delay in the amplification of the longer isoform (e14a2) (median Tt: 18.48 vs. 26.08 min, respectively; *p* < 0.001). A median time of 19.63 was observed in samples expressing both p210 isoforms (e13a2/e14a2) (Table 2). This value was not significantly different than the Tt observed in samples with e13a2 (*p* = 0.1332) (Figure 2A). On the contrary, a statistically significant difference was detected between samples with both e13a2 and e14a2 and patients carrying the e14a2 *BCR-ABL1* transcript alone (*p* < 0.001) (Figure 2A). In order to verify if we could define a cutoff in Tt minutes that would predict the expression of the common p210 variants, we performed a ROC analysis, clustering e13a2 and e13a2/e14a2 patients as no differences in Tt were observed between these groups. This analysis showed that a Tt higher than 21.55 min may be associated with a higher probability of identifying e14a2-positive patients with a sensitivity of 88.37% and a specificity of 95.38% (Figure 2B). 

### 2.3. Concordance between the Q-LAMP Assay and the Standard BIOMED-1 Method on Rare p210 and p190 BCR-ABL1 Isoforms

Approximately 5% of all CML or ALL patients are diagnosed with rare *BCR-ABL1* rearrangements that involve fusion of alternative exons, insertions, or breakpoints within exons such as e19a2 (the most common rearrangement), e8a2, e13a3, e14a3, e1a3, and e6a2. 

In this study, we analyzed 20 patients with rare *BCR-ABL1* isoforms (5 with e1a3, 9 with e13a3, 3 with e14a3 and 3 with e19a2) (Table 3). Although the Q-LAMP BCR-ABL qualitative assay is not designed to detect or discriminate rare chromosomal rearrangements, the primer sets have been improved to potentially amplify some of them. Interestingly, we obtained a 100% concordance rate between standard RT-PCR and the Q-LAMP assay. The Tts observed were higher compared to the common isoforms (Table 3), probably due to the longer transcripts deriving from the presence of additional exons (Table 3).

## 3. Discussion

Q-LAMP is a qualitative assay for the detection and the discrimination of chromosomal translocations performing simultaneous RNA reverse transcription and DNA amplification. Previously published data generated from serial dilutions of RNA derived from ALL or CML cell lines (TOM-1 or K-562) demonstrated that the Q-LAMP assay detected and discriminated the p190 (e1a2) and p210 (e13a2 and e14a2) *BCR-ABL1* transcripts within 50 min. The assay amplifies these different targets, including the endogenous internal control *GUSβ* gene [33], in a single tube and in a single step. The authors also demonstrated that Q-LAMP showed 100% specificity on 70 replicates of wild type RNA from 7 *BCR-ABL1*-negative cell lines [33].

In order to evaluate if this technology may represent a valid alternative to the qualitative BIOMED-1 PCR for CML and ALL patients, we analyzed a total of 122 *BCR-ABL1*-positive and 50 negative samples using the Q-LAMP assay. In our analysis, the Q-LAMP showed complete concordance with the BIOMED-1 PCR with a significantly lower time-to-results ratio, allowing the identification of *BCR-ABL1*-positive patients in about 30 min (Table 2). Additionally, in the Table 4, we compared the two techniques with emphasis on the advantages of each approach. 

The molecular detection of *BCR-ABL1* fusion transcripts is a necessary step for the diagnosis and risk classification of CML and ALL [1,2]. In the current clinical setting, the introduction of multiple TKIs has generated unprecedented rates of hematological, cytogenetic, and molecular responses, increasing disease-free survival and overall survival [34,35]. Different studies reported that the e13a2 isoform is associated with lower rates of complete cytogenetic response and major and deep molecular response to different TKI [10,15,16,19]. Furthermore, recent studies have found that the e13a2 transcript has a significant adverse impact on the maintenance of a treatment-free remission (TFR) after stopping TKI therapy [36,37]. Therefore, it is essential to correctly identify the *BCR-ABL1* isoforms expressed by each patient. These molecular analyses should be performed by a specialized center that is routinely monitored by a reference laboratory. In our study, we found that the Q-LAMP assay distinguished the *BCR-ABL1* e13a2 variant from the e14a2 isoform (median Tt 18.48 vs. 26.08 min, respectively; *p* < 0.001). A recent study by Baccarani et al. reported that the percentage of patients co-expressing both e13a2 and e14a2 isoforms ranges between 1.1% and 26.9% [38]. We found a statistically significant difference between the Tt in samples co-expressing e13a2/e14a2 and those with the e14a2 isoform alone (Figure 2). On the contrary, we observed only a 1 min Tt difference between specimens expressing e13a2 or co-expressing e13a2/e14a2. We assume that the observed Tt differences are influenced by the amplification of different isoforms and that the e13a2 amplification may have an advantage compared to e14a2, probably due to the shorter length of the former transcript. Therefore, when the Q-LAMP assay shows an amplification range compatible with the e13a2 transcript or with the co-existence of both e13a2/e14a2, a confirmatory RT-PCR may be warranted following the BIOMED-1 protocol [25]. 

Approximately 5% of Ph-positive patients are diagnosed with rare *BCR-ABL1* rearrangement. Although the purpose of the Q-LAMP assay is the detection and discrimination of the most common *BCR-ABL1* transcripts, the primer sets have been improved in order to potentially detect rare oncogenic isoforms. In this study, we observed 100% concordance rates between the Q-LAMP and the standard BIOMED-1 method when analyzing infrequent isoforms. We hypothesize that the observed delay in term of Tt of these rare isoforms might be due to the presence of additional exons, which encode for longer transcripts. Based on the results of our study, Q-LAMP might represent a valid option for the reliable and rapid detection of rare *BCR-ABL1* fusion. However, further studies on larger populations should be performed in order to confirm our findings.

In conclusion, the Q-LAMP assay satisfies all the safety requirements of a validated diagnostic test coupled with being a straightforward technical approach, thanks to the pre-assembled lyophilized reagents and a one-step procedure that limits the risks of errors and optimizes laboratory workflow. This closed-tube format could easily be adopted in non-highly specialized centers for an effective diagnosis of Philadelphia-positive leukemias.

As an increasing number of manuscripts [10,15,16,19] suggest that in the future, the *BCR-ABL1* variant may be included in the calculation of baseline risk scores, it is possible to envision a scenario in which rapid and accurate determination of the *BCR-ABL1* transcript will aid physicians in defining which patients will achieve greater benefit from the different available TKIs.

## 4. Materials and Methods

### 4.1. Patients

Bone marrow (BM) and peripheral blood (PB) samples were collected from 142 patients with newly diagnosed chronic phase CML and Philadelphia-positive ALL. Patients were followed in four Centers: Laboratorio di Medicina Interna ad Indirizzo Ematologico, Azienda Ospedaliera Universitaria San Luigi Gonzaga (Turin); Laboratorio di Ematologia Oncologica, Ceinge-Biotecnologie Avanzate, Università di Napoli “Federico II” (Naples); Centro di Oncologia ed Ematologia Sperimentale, Azienda Ospedaliera Universitaria “Policlinico-Vittorio Emanuele” (Catania), and the Molecular Biology Unit, University Hospital La Fe (Valencia).

The samples were collected from January 2016 to May 2019 and were retrospectively analyzed based on the results previously obtained with the BIOMED-1 protocol [25]. The total analyzed RNA was derived from 14 samples with p190 (e1a2), 87 samples with the p210 transcript (44 samples with e13a2 and 43 samples with e14a2), and 21 samples co-expressing both e13a2 and e14a2 (Supplementary Table S1). Additionally, the analysis was performed on 20 samples derived from CML and ALL patients with rare isoforms (5 with e1a3, 9 with e13a3, 3 with e14a3, and 3 with e19a2) (Supplementary Table S1). Fifty negative samples were also included in the study, 24 from *BCR-ABL1*-negative patients and 26 from healthy donors. 

Informed consent was obtained from each subject, and all procedures were performed in accordance with the Helsinki Declaration and subsequent revisions.

### 4.2. RNA Extraction

Total RNA was extracted from PB or BM polymorphonuclear cells collected in EDTA or sodium citrate, using different methods: phenol–chloroform (N = 7), RNeasy Mini Kit, QIAGEN (N = 50), QIAsymphony, QIAGEN (N = 72), and Maxwell 16 LEV simplyRNA Kit, Promega (N = 63), according to the respective manufacturers’ instructions.

Purified total RNA was quantified with a spectrophotometer (NanoDrop, Technologies Inc.) at wavelengths of 230, 260, and 280 nm. RNA integrity was then verified by running samples on 1.2% denaturing agarose gels. Total RNA samples were stored at −20 or −80 °C until their use.

### 4.3. BCR-ABL1 Q-LAMP Assay

**Figure 3:**
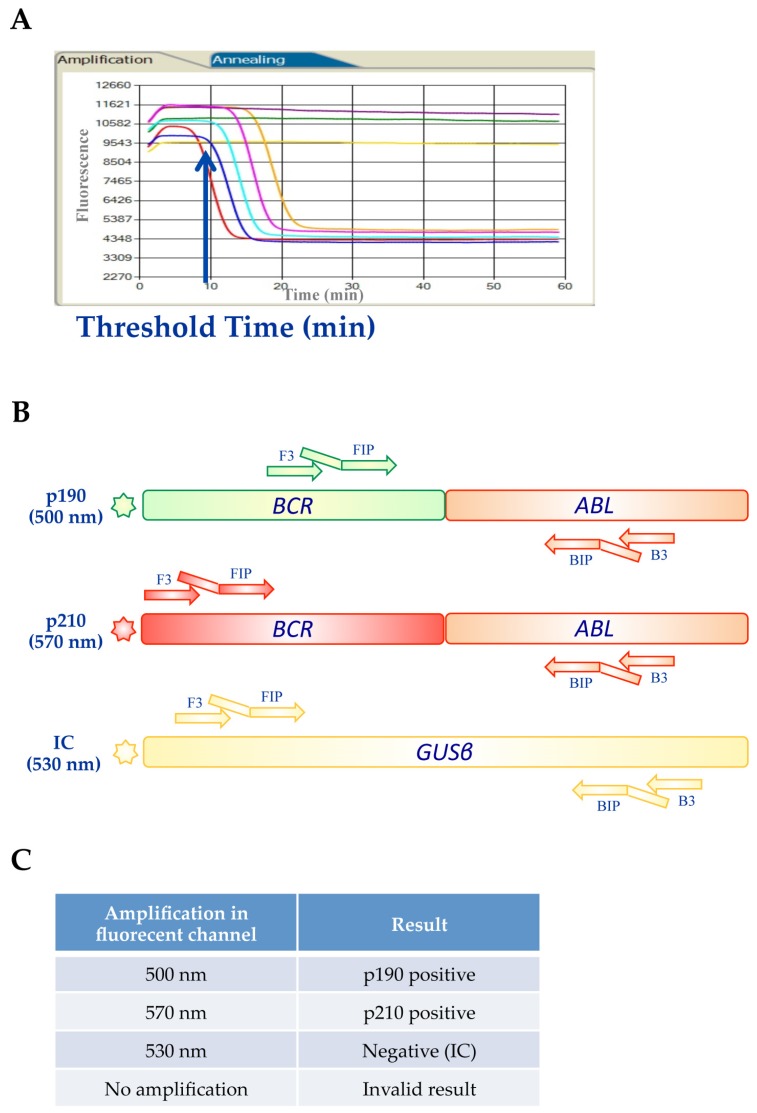
*BCR-ABL1* Q-LAMP Assay. (**A**) Representative amplification curves for *BCR-ABL1*-positive samples. (**B**) Design with indicated oligonucleotide primers of the triplex fluorescent Q-LAMP assay that detects and discriminates the *BCR-ABL1* p190 (e1a2) and p210 (e13a2, e14a2) transcripts. (**C**) *BCR-ABL1* p190-positive samples generate a signal in the 500 nm channel, while samples positive for p210 generate a signal in the 570 nm channel. The amplification of the *GUSß* control gene produces a signal in the 530 nm channel.

The Q-LAMP is a qualitative assay for the detection and the discrimination of chromosomal translocations directly from total RNA samples. The original LAMP method has been modified introducing a polymerase with simultaneous RNA reverse transcription and DNA amplification activity. The implementation of fluorescent dyes allowed to perform multiplex reaction analyses by using specific primer sets. The assay includes an endogenous housekeeping gene (*GUSß*) that is amplified as an internal control in the same tube of the target reaction to check for the real absence of the translocation or for artifacts such as RNA degradation, reaction inhibition, or technical failure. The fluorescent signals can be monitored in real time onto the dedicated instrument LIAISON^®^ IAM (DiaSorin S.p.A., Saluggia, Italy) that provides results within 60 min. At time zero, the reaction excited with appropriate wavelength UV light emits maximum fluorescence. As a consequence of the amplification, fluorescence decreases exponentially thanks to the natural quenching effect of newly synthetized amplicons (Figure 3A).

The *Iam* BCR-ABL qualitative assay is a triplex fluorescent Q-LAMP assay designed for the differential detection and discrimination of *BCR-ABL1* p190 (e1a2) and p210 (e13a2, e14a2) fusion transcripts in a single one-step reaction with pre-assembled lyophilized reagents (Figure 3B).

A total of 500 ng of RNA was used for each sample in a final reaction volume of 25 μL. The assay was performed following the manufacturer’s instructions (DiaSorin S.p.A., Saluggia, Italy) and each analytical session included a p190/p210 double-positive control, a negative control (positive for the housekeeping *GUSß* gene), and a no template control (nuclease-free water). During the amplification, *BCR-ABL1* samples positive for p190 generate a signal in the 500 nm channel, while samples positive for p210 generate a signal in the 570 nm channel. All *BCR-ABL1*-negative samples were confirmed from the amplification of the *GUSß* control gene that produces a signal in the 530 nm channel (Figure 3C). The housekeeping *GUSß* gene was not amplified in *BCR-ABL1* p190 (e1a2) or p210 (e13a2, e14a2) samples, due to the preferential amplification of the oncogenic isoforms. On the contrary, samples harboring the rare *BCR-ABL1* p190 (e1a3) or p210 (e13a3, e14a3 and e19a2) isoforms were amplified at a later time, with a positive signal for the *GUSß* control gene.

All results were expressed as threshold time (Tt) in minutes, defined as the minute at which the sample fluorescence reaches 50% of quenching [30].

### 4.4. Statistical Analyses

Threshold times of the three different p210 isoform groups (e13a2, e14a2 and e13a2/e14a2) were compared using the Kruskal–Wallis test followed by Dunnett’s multiple comparisons. The variance of the groups was calculated with the Bartlett test and the data distribution with the Kolmogorov–Smirnov test. The receiver operating characteristic (ROC) approach was applied to identify a possible cutoff in terms of Tt between carriers of different isoforms of the p210 fusion gene. Sensitivity and specificity were calculated, and the curve and the area under the curve (AUC) were defined. All analyses were performed using GraphPad Prism version 8.1.1 for Windows (GraphPad Software, La Jolla California USA, www.graphpad.com).

## Figures and Tables

**Table 1:** Concordance between the Q-LAMP assay and the standard BIOMED-1 method on common *BCR-ABL1* isoforms.

		Results by Conventional RT-PCR (BIOMED-1)			
		p190	p210	Negative	Total
**Q-LAMP Assay**	p190	14	/	/	14
	p210	/	108	/	107
	Negative	/	/	50	50
	Total	14	108	50	172

**Table 2:** Q-LAMP performance using common *BCR-ABL1* isoforms.

Isoform (N Sample)	Mean Tt * (st. dev)	Median Tt * (Range)	CV
**e1a2**	27.03	26.70	0.07
(14)	(1.87)	(24.45–31.80)	/
**e13a2**	18.64	18.48	0.08
(44)	(1.41)	(15.25–21.67)	/
**e14a2**	25.46	26.08	0.13
(43)	(3.29)	(16.53–34.57)	/
**e13a2/e14a2**	19.84	19.63	0.06
(21)	(1.29)	(17.93–23.20)	/

* Threshold times (Tt) are reported in minutes; CV = coefficient of variation.

**Table 3:** Concordance between the Q-LAMP assay and the reference BIOMED-1 method on rare p190 and p210 *BCR-ABL1* isoforms.

		Results by conventional RT-PCR (BIOMED-1)				
		e1a3	e13a3 (b2a3)	e14a2 (b3a3)	e19a2	Total
**Q-LAMP Assay**	e1a3	5	/	/	/	5
	e13a2 (b2a3)	/	9	/	/	9
	e14a2 (b3a3)	/	/	3	/	3
	e19a2	/	/	/	3	3
	Total	5	9	3	3	20

**Table 4:** Comparison of the Q-LAMP assay and the BIOMED-1 method.

	Q-LAMP Assay	BIOMED-1 Method
**Nucleic acid input (RNA ng/sample)**	RNA (500)	cDNA (1000)
**Number of steps**	1	≥3
**Reaction setup**	Easy	Moderately complex (RT-PCR is required)
**Detection of rare *BCR-ABL1* isoforms**	Possible	Possible
**Time to results (minutes)**	<60	>300
**Mark**	CE/IVD	LDT

RT-PCR = reverse transcriptase PCR; LDT = laboratory developed test.

## Supplementary Materials

Supplementary materials can be found at https://www.mdpi.com/1422-0067/20/24/6106/s1.

## Abbreviations

ABL1	Abelson murine leukemia
ALL	acute lymphoblastic leukemia
BCR	breakpoint cluster region
CML	chronic myeloid leukemia
GUSβ	β-glucuronidase
LAMP	loop-mediated isothermal amplification
ROC	receiver operating characteristic
TK	tyrosine kinase
TKI	tyrosine kinase inhibitor
TFR	treatment-free remission
