# Supplementary material for: The Q-LAMP Method Represents a Valid and Rapid Alternative for the Detection of the BCR-ABL1 Rearrangement in Philadelphia-Positive Leukemias
Source: Int J Mol Sci. 2019 Dec 4;20(24):6106. doi: 10.3390/ijms20246106 (PMC6941015; doi:10.3390/ijms20246106)
Supplement: Supplementary file 1 [file ijms-20-06106-s001.pdf]

**Supplementary Table 1. Patient Characteristics at the time of diagnosis (N 142)**

| Characteristics | | % |
|---|---|---|
| **Age (years)** | | |
| Median | 60.32 | |
| Range | 20-89 | |
| **Sex** | | |
| Male | 91 | 64 |
| Female | 51 | 36 |
| ***BCR-ABL1* Isoform** | | |
| e1a2 | 14 | 9.9 |
| e13a2 | 44 | 31.0 |
| e14a2 | 43 | 30.3 |
| e13a2 - e14a2 | 21 | 14.8 |
| e1a3 | 5 | 3.5 |
| e13a3 | 9 | 6.3 |
| e14a3 | 3 | 2.1 |
| e19a2 | 3 | 2.1 |
